# Tailoring and evaluating the web-based ‘Partner in Balance’ intervention for family caregivers of persons with young-onset dementia

**DOI:** 10.1016/j.invent.2021.100390

**Published:** 2021-04-23

**Authors:** Jeroen Bruinsma, Kirsten Peetoom, Christian Bakker, Lizzy Boots, Joany Millenaar, Frans Verhey, Marjolein de Vugt

**Affiliations:** a Department of Psychiatry and Neuropsychology/Alzheimer Centre Limburg, School for Mental Health and Neuroscience, Maastricht University, Maastricht, the Netherlands; b Department of Primary and Community care, Radboud University Medical Centre, Nijmegen, the Netherlands; c Radboudumc Alzheimer Centre, Nijmegen, the Netherlands; d Groenhuysen, Centre for Specialized Geriatric Care, Roosendaal, the Netherlands; e Laurens Residential Care, Rotterdam, the Netherlands

**Keywords:** YOD, young-onset dementia, FTD, frontotemporal dementia, AD, Alzheimer's dementia, Young-onset dementia, Caregiver, eHealth, Psychosocial support, Intervention, Tailoring

## Abstract

Young-onset dementia (YOD) poses specific challenges for caregivers involved. However, most available support does not address their specific needs. Previously, the web-based Partner in Balance intervention showed promising results and facilitated role adaptation in dementia caregivers. Although the web-based format proved a good fit for YOD caregivers, the evaluation showed a need for tailored content on YOD. Therefore, new content was iteratively developed respectively for spouses and other family caregivers of persons with YOD. This study evaluates how caregivers perceived the tailored content.

**Methods:**

A pre-post design was used to prospectively evaluate how end-users perceived two tailored versions of the Partner in Balance intervention, one for spouses and one for other family members of people with YOD. After the intervention, participants were interviewed for approximately 60 min in-person or by telephone using the Program Participation Questionnaire. A qualitative deductive content analysis was used to evaluate (1) usability, (2) feasibility and acceptability, (3) perceptions on intervention content. To evaluate if the intervention facilitated role adaptation, preliminary effects were examined using pre-post questionnaires on self-efficacy, mastery, stress, anxiety and depression.

**Results:**

Spouses (*n* = 11) and other family members (*n* = 14) both positively evaluated the tailored content on YOD and valued that the web-based approach could easily be integrated in daily life. Participants perceived the intervention as usable, feasible and acceptable. Participants valued the recognizability of the content. Goal-setting helped participants to translate the intervention to daily life, although for some participants setting goals was difficult. Caregivers of persons with frontotemporal dementia suggested incorporating specific content to further increase recognizability. After participation, participants felt better equipped for the caregiving role. In line with previously demonstrated effects on generic modules of Partner in Balance, the tailored version increased levels of self-efficacy in the group of other family caregivers, *t*(12) = 3.37, *p* = .006.

**Conclusion:**

The tailored Partner in Balance intervention was positively evaluated by YOD caregivers. Offering participants more guidance on goal-setting and adding content about frontotemporal dementia may facilitate implementation.

## Highlights

• Partner in Balance fits the needs of young-onset dementia caregivers.
• Tailored content showed to be usable, feasible, and acceptable.
• Caregivers felt the web-based approach could easily be integrated in daily life.
• Partner in Balance facilitated role adaptation and coping with future challenges.
• Incorporating tailored content on frontotemporal dementia is a future direction.

## 1 Introduction

Symptoms of young-onset dementia (YOD) start before the age of 65 and pose specific challenges, not only to the person with YOD but also to the family members involved (Cabote et al., 2015; Rossor et al., 2010). Due to the young age at onset, spouses often combine caregiving with employment (Caceres et al., 2016; van Vliet et al., 2010b), making it difficult for the caring partner to balance care related responsibilities and family life (Kilty et al., 2019; Roach et al., 2013). Children may still live at home and gradually become caregiver for their parent with YOD (Millenaar et al., 2014). This may have negative long-term effects on the future of children. For example, because they postpone plans regarding studying or moving out of the parental house (Millenaar et al., 2014). Family members often experience difficulty in coping with the profound changes in personality and behavior in their relative with YOD and feel uncertain about the future. This is known to cause high levels of burden and distress in family members (Cabote et al., 2015; van Vliet et al., 2010b). Additionally, family members are known to experience a shift in their social role as they become increasingly responsible for their relative with YOD (Hutchinson et al., 2016; Richardson et al., 2016). The ability to adapt to the role of caregiver is complicated by specific characteristics of YOD that prolong the time to diagnosis. The time to diagnosis is 4.4 years in YOD while in late onset dementia this is 2.8 years (van Vliet et al., 2013). This diagnostic delay impedes role adaptation because a diagnosis helps family members to understand the changes in their relative and to see the caregiving role in perspective (de Vugt and Verhey, 2013).

Psychosocial support may help caregivers to come to terms with their new role (de Vugt and Verhey, 2013; Kilty et al., 2019; Millenaar et al., 2018). However, most available support is targeted at spouses of persons with YOD or caregivers of older persons with dementia (Cations et al., 2017; Millenaar et al., 2016a). Given the impact of YOD on family life it is indicated that support should target the family as a whole (Chapman et al., 2019; Hutchinson et al., 2016; Kaizik et al., 2017; Kilty et al., 2019). Supporting the whole family may create a sense of togetherness and this may empower the caregiving system by making caregivers more resilient (Cabote et al., 2015; Roach et al., 2013). As all family members have their unique support needs (Millenaar et al., 2014), a tailored approach seems required to adequately support spouses and other family members of persons with YOD. Given the low prevalence of YOD and the active life phase of the family members involved, web-based support tools may provide an opportunity to facilitate accessible support (Diehl-Schmid et al., 2013; Harvey et al., 2003). However, web-based support for family members of persons with YOD is scarce or primarily aimed at psycho-education (Kaizik et al., 2017; Kurz et al., 2016; Nichols et al., 2013). To facilitate role adaptation, more attention is needed for support that helps family members cope with daily challenges.

The web-based Partner in Balance intervention uses self-management principles to facilitate role adaptation in caregivers by increasing levels of self-efficacy, sense of mastery, and the quality of life in caregivers (Boots et al., 2018; Boots et al., 2016). In Partner in Balance, caregivers watch video vignettes, read background information, make self-reflection assignments and set goals for the future together with a personal coach. Currently, efforts are made to implement the intervention (Christie et al., 2020). The web-based format showed to be a good fit for caregivers of persons with YOD as they are more likely to participate due to the online nature, compared to older caregivers (Boots et al., 2018). Despite this, a process evaluation revealed that caregivers of persons with YOD missed specific content on YOD in the Partner in Balance intervention, as most original content portrays elderly (Boots et al., 2017). Therefore, new tailored content was separately developed for spouses and other family caregivers of persons with YOD in order to be incorporated in the intervention. This study reports the results of two end-user tests regarding these tailored versions of the Partner in Balance intervention. Based on previous research (Boots et al., 2018; Boots et al., 2016), we hypothesized that the tailored Partner in Balance intervention would provide a good fit in terms of usability, feasibility and acceptability. Additionally, we hypothesized that participants would report higher levels of self-efficacy and mastery, and lower levels of stress, anxiety and depression after the intervention.

## 2 Methods

This feasibility study used a pre-post design to prospectively evaluate how end-users perceive newly incorporated content in the Partner in Balance intervention in terms of usability, feasibility, acceptability, and perceptions on content (Aggarwal and Ranganathan, 2019).

### 2.1 The Partner in Balance intervention

**Fig. 1:**
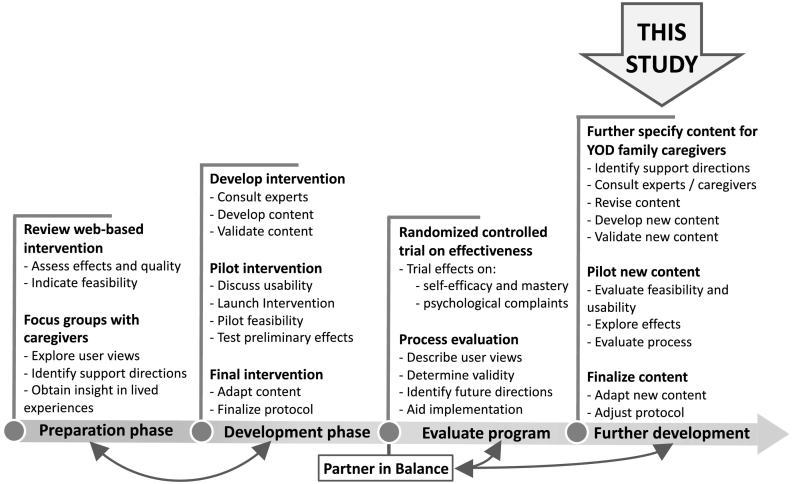
Development of the Partner in Balance intervention.

The web-based Partner in Balance intervention is an effective and feasible support tool for family caregivers of persons with dementia who still live at home (Boots et al., 2018). The intervention has been iteratively developed using a stepwise approach guided by the Medical Research Council (MRC) framework for developing and evaluating complex interventions (Fig. 1) (Boots et al., 2016; Craig et al., 2008). The intervention incorporates self-management principles to help caregivers find a balance between caregiving and daily life (Boots et al., 2017). Caregivers follow the intervention individually and receive personal online coaching from a trained healthcare professional while they subsequently follow four self-chosen thematic modules online (Table 1). The duration of Partner in Balance is approximately eight to ten weeks but this is flexible (Boots et al., 2017). Each module includes (1) a video vignette in which caregivers share their experiences about a specific theme, (2) psychoeducation including a narrative story and practical tips, (3) a self-reflection assignment, and (4) a step-by-step change plan (Boots et al., 2016). Per module, the coach provides feedback to support caregivers with reflecting on their situation, and to formulate specific and attainable goals. Using a built-in chat, caregivers are also able ask questions directly to the coach. After completing four modules, caregivers reflect on their personal development together with the coach. On average, coaches spend around 6 h during eight weeks to supervise caregivers using Partner in Balance (Boots et al., 2017).

**Table 1:** Incorporated modules in the Partner in Balance intervention.

Modules	Original modules	New modules for YOD caregivers	
	Boots et al., 2016	Spouses	Other family members
Combining care with work		x	x
Impact on family life		x	x
Sexuality and intimacy		x	
Worries about heredity			x
Coping with stress^a^	x		
Acceptance	x	x	x
Balance in activities	x	x	x
Changes in relative with YOD	x	x	x
Communication	x	x	x
Focusing on the positive	x	x	x
Insecurities and rumination	x	x	x
Self-understanding	x	x	x
Social relationships and support	x	x	x

a The module on coping with stress was merged with focusing on the positive and insecurities and rumination.

#### 2.1.1 Incorporating new content for YOD caregivers in the Partner in Balance intervention

After the effect evaluation, a process evaluation revealed that the Partner in Balance intervention needed specific tailoring for spouses and other family caregivers (e.g. children, brothers or sisters, and parents) of persons with YOD (Boots et al., 2018; Boots et al., 2017). New intervention content was iteratively developed using the MRC-framework. First, experiences and needs of YOD caregivers were identified from the literature and derived from the results of the NEEDs in Young onset Dementia (NeedYD) study (Bakker et al., 2013; van Vliet et al., 2010a). Second, directions for adaptation of the intervention were verified by consulting field experts (researchers, a neurologist, a clinical geneticist), healthcare professionals (dementia casemanagers, psychologists), and family members of persons with YOD. All original intervention content was critically revised to fit the specific needs of YOD caregivers. This resulted in the development of module content specific for either spouses or other family members of persons with YOD. Additionally, four new modules were developed covering (1) the impact of dementia on family life, (2) combining caregiving tasks with work or school, (3) sexuality and intimacy, and (4) worries about heredity (see Table 1). New video vignettes were developed by conducting in-depth interviews with four spouses and three other family members (i.e. two children and a sister) of a person with YOD. The interviews also allowed validating and supplementing the newly developed module content. Subsequently, a draft version was put online and feedback was asked to field experts, healthcare professionals, spouses, and other family members of persons with YOD. This resulted in two separate versions of the Partner in Balance intervention; one for spouses and one for other family members. Two end-user tests were conducted, to evaluate both versions. In the end-user tests participants followed four tailored modules in conjunction with receiving online coaching. All participants were interviewed afterwards to evaluate the newly developed modules and completed a questionnaire before and after the intervention to obtain insight in preliminary effects on role adaptation.

### 2.2 Recruitment of participants

Spouses and other family members were recruited for the end-user tests by (1) spreading information within the network of Alzheimer Centre Limburg/MUMC+, (2) providing information about the study in bimonthly meetings with healthcare providers affiliated with the Dutch Young-onset Dementia Knowledge Centre [*Kenniscentrum Dementie op Jonge Leeftijd*], and by (3) distributing information via social media and meetings of the Dutch peer-support organization [*FTD lotgenoten*]. Spouses and other family caregivers contacted the first author if they wanted to participate. A previous study demonstrated that the inclusion of ten participants was sufficient to evaluate generic modules of Partner in Balance (Boots et al., 2016). Other feasibility studies on interventions for dementia caregivers included smaller sample sizes (Fick et al., 2011; Lai et al., 2012). Due to the heterogeneity of YOD we aimed to include ten spouses and 15 other family caregivers in our end-user test. Participants were eligible for participation if (1) they were aged 16 years or older, (2) they had a spouse or other relative with dementia with a symptom onset before the age of 65, and (3) their relative with dementia still lived at home.

During the end-user test, participants received online coaching (Boots et al., 2017). Therefore, psychologists and dementia casemanagers (specialized nurses or social workers) were recruited to become a coach. Preferably, these healthcare professionals would coach a caregiver from their own caseload. Healthcare professionals were eligible when they were employed as a dementia casemanager or psychologist and had practical experience with supporting YOD caregivers. At the start of the intervention, all healthcare professionals received a two-hour training to get acquainted with Partner in Balance, principles of self-management, and elements of web-based support (Boots et al., 2016). Following suggestions of Christie et al. (2021), a low threshold was pursued for technological support and intervision with more experienced coaches. Therefore, the first author contacted the coaches on a bi-weekly basis to monitor the process and evaluate if they encountered any difficulties. Also, intervision was organized between experienced and less experienced coaches. In addition to external healthcare professionals, coaching was also performed by five coaches of the Alzheimer Centre Limburg with a background in psychology and extensive experience with coaching in Partner in Balance.

### 2.3 Measurements used during the end-user test

In line with previous studies on the Partner in Balance intervention (Boots et al., 2018; Boots et al., 2016), qualitative and quantitative measures were used to evaluate the newly developed content.

#### 2.3.1 The Program Participation Questionnaire

To obtain insight in how participants perceived the intervention, semi-structured interviews were conducted after the intervention by means of the Program Participation Questionnaire (PPQ) (Boots et al., 2016). The PPQ consists of 33 items covering (1) how the intervention was used and implemented in daily life, (2) if the intervention was feasible, usable and acceptable, and (3) how the quality and quantity of the content was perceived. Each item can be scored on a Likert scale ranging from 1 “strongly disagree” to 7 “strongly agree”. To obtain insight in participant reflections and experiences with the intervention, caregivers were encouraged to elaborate on their scores during the interview, while the researchers made fieldnotes. The PPQ also examines role adaptation by asking if the intervention positively influenced coping and if caregivers felt more confident towards their role as a caregiver.

#### 2.3.2 Analysis of the Program Participation Questionnaire

The results of the PPQ were first analyzed quantitatively by calculating descriptive statistics such as mean, range, and percentiles. Items with a mean lower than 5 “slightly agree” were considered as a direction for improvement. To estimate the overall feasibility, usability and acceptability, a total PPQ-score was calculated ranging between 33 and 231 (Median = 132). In line with previous studies (Boots et al., 2016; Campbell et al., 2012), the median was used as a cut-off score to determine overall feasibility, usability, and acceptability. Subsequently, a deductive qualitative content analysis was performed using fieldnotes to interpret the quantitative scores on the PPQ (Elo and Kyngäs, 2008). A pragmatic theoretical stance was used to evaluate if the intervention matched the needs of the target group (Morgan, 2014). Therefore, the first author deductively coded and classified all fieldnotes in five categories focusing on the (1) use of the intervention in daily life, (2) feasibility, usability and acceptability, (3) quantity and quality of the content, (4) role adaptation and well-being, and (5) suggestions for improvement and directions for the future. The coded and categories were discussed in a consensus meeting with the second author, after which the findings were also discussed with the wider research team to verify and substantiate the findings.

#### 2.3.3 Preliminary effects on role adaptation

Additionally, we aimed to evaluate if the effects of the tailored versions were in line with those of the previously conducted feasibility study and randomized control trial (Boots et al., 2016; Boots et al., 2018). Therefore, a set of questionnaires was composed to examine preliminary effects and evaluate if the tailored versions of the Partner in Balance intervention facilitated role adaptation. Similar to earlier studies, questionnaires covered both self-efficacy and mastery. Self-efficacy was assessed with the Self-Efficacy Scale (CSES) (Fortinsky et al., 2002). Items covered care-management (six items) and service use (four items) and could be scored from 1 “not at all” to 10 “very”. Sense of mastery was assessed with the Pearlin Mastery Scale (PMS) (Pearlin and Schooler, 1978). Items covered sense of control and problem solving ability, using seven items that could be scored between 1 “not at all” to 5 “totally agree”.

Role adaptation may have a positive effect on psychological well-being (Bandura, 1997; Boots et al., 2018; Boots et al., 2016). The questionnaire therefore also contained ten items of the Perceived Stress Scale (PSS) (Cohen, 1988). Items examined perceived levels of stress during the past seven days and were scored between 0 “never” to 4 “very often”. The Hospital Anxiety and Depression Scale (HADS) was also included (Zigmond and Snaith, 1983). Items assessed levels of anxiety (six items) and depression (seven items) and could be scores from 0 “not at all” to 3 “often”.

#### 2.3.4 Analysis of the preliminary effects on role adaptation

To evaluate preliminary effects, the averaged questionnaire scores before and after the intervention were compared. Paired-sample t-testing was used to assess if any significant effects occurred. All analyses were conducted in SPSS (version 25.0) using an alpha level of 0.05 for two-sided tests.

### 2.4 Ethical considerations

The study protocol was approved by the Medical Ethics Committee of Maastricht University Medical Centre, the Netherlands (METC: 2018-0443). Before inclusion, all participants received a letter including information about the study protocol, data-security and privacy by email and were phoned to see if they had any additional questions. In advance to enrolling the end-user test, all participants completed a consent form.

## 3 Results

**Fig. 2:**
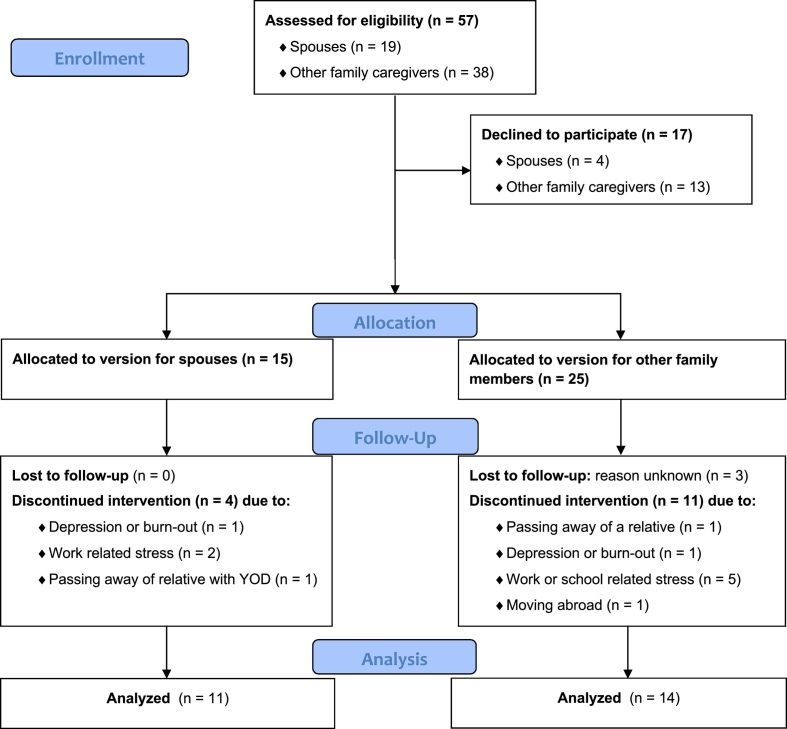
CONSORT flow diagram on participation in the end-user tests (Moher et al., 2001).

Eventually, 15 out of 19 (78.9%) spouses and 25 out of 38 (65.8%) other family members (i.e. 21 children, three siblings, and one parent) gave consent and participated in the end-user tests (Fig. 2). Alzheimer's dementia (AD) (*n* = 27) was the most prevalent cause, followed by frontotemporal dementia (FTD) (*n* = 9), vascular dementia (*n* = 3), and Lewy Body dementia (*n* = 1). The majority of participants received coaching from a coach of the Alzheimer Center Limburg, while six spouses and six other family members received coaching from their casemanager or psychologist. The end-user test was completed by 11 of the 15 (73.3%) spouses, and 14 of the 25 (56.0%) other family members (Table 2). According to the non-completers their reason to withdraw from participation was not related to the intervention but to important life events such as the passing away of their relative with YOD (*n* = 1) or another relative (*n* = 1), moving abroad (*n* = 1), depression (*n* = 2), work or school related stress (*n* = 7), or unknown (*n* = 3). The non-completers did not significantly differ from the completers on demographic characteristics or outcome variables.

**Table 2:** Characteristics of included participants.

Caregiver	N (%)	Male/female	Age (min-max)
Completers			
Spouse	11 (45.8)	4/7	58.7 (50–70)
Child	9 (33.3)	3/6	33.5 (18–44)
Sibling	4 (12.5)	1/3	59.8 (56–64)
Parent	1 (4.2)	0/1	74.0
Non-completers (*n* = 15)			
Spouse	4 (26.6)	3/1	58.3 (53–65)
Child	11 (73.3)	4/7	30.9 (17–45)

### 3.1 The Program Participation Questionnaire

**Fig. 3:**
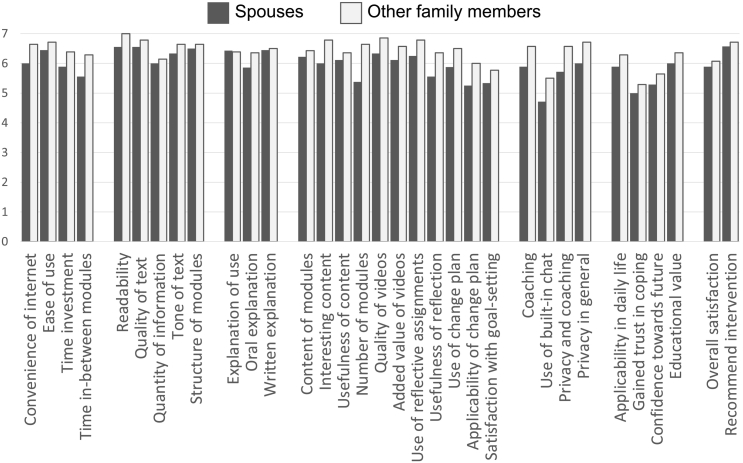
Averaged scores on the 33-item Program Participation Questionnaire.

After completing the intervention, the average total score on the PPQ was 195.8 (SD = 15.8) for the 11 spouses. The total score for the 14 other family members was 211.3 (SD = 13.1). Both scores are higher than the cut-off score of 132, indicating a good overall usability, feasibility, and acceptability. On average, the spouses scored all items higher than 5 “slightly agree”, except for the item covering the use of the chat function (Fig. 3). Percentiles revealed that 25% of the spouses scored the use of the chat and goal-setting lower than 5 “slightly agree”. The end-user test with other family members showed that all averaged scores and 25% percentiles were higher than 5 “slightly agree”, indicating good feasibility.

In line with the scores on the PPQ, a deductive qualitative content analysis of the fieldnotes showed that both spouses and other family members valued the intervention for its usability in daily life. They particularly appreciated the web-based format because it allowed them to follow the intervention in their own time at home. This facilitated the use of the intervention as most participants combined caregiving with work or studying, family life, and social activities. Spouses and other family members described the lay-out as self-explanatory, well structured, and user friendly. Particularly children (aged 18 or older), felt it was easier for them to express their feelings online, compared to traditional in-person meetings with healthcare professionals. Some children suggested to further improve the usability by making the intervention compatible with smartphone usage to allow them to use it in public transport while commuting to school or work.

“The internet component was nice because you can easily share your feelings. […] I was able to share things outside the presence of my parents.”

– 17 year old son of a person with frontotemporal dementia –

The quality of the tailored content was appreciated and considered highly acceptable by both spouses and other family members. They valued the recognizability of the video vignettes and narrative stories as this made them feel recognized. All participants felt that the written materials were well articulated, complete, and recognizable. However, some spouses and other family members of persons with FTD explained having difficulty recognizing themselves in the personal stories, which mainly focused on AD. They suggested to incorporate more specific content about FTD in the intervention to improve recognizability.

“The videos make you feel recognized and understood. They also tackle the taboo to talk about dementia.”

– 44 year old daughter of a person with Alzheimer's dementia –

Spouses and other family members also valued the self-reflection assignments as it helped to translate the intervention to their personal situation. The reflection assignments helped to critically think about their needs and helped to prioritize activities. Most participants also perceived the step-by-step change plan as an important element because it structured their way of thinking by offering a stepwise approach to work towards a personal goal. However, some participants in both groups struggled with the step-by-step change plan experiencing difficulty formulating specific, measurable and attainable goals. The feedback from the coach was experienced as an important source of inspiration while setting goals as it helped participants to gain new insights.

“The coach is the most important part of Partner in Balance. The personal contact is very nice and the coach helps you to set goals by giving advice.”

– 64 year old brother of a person with Lewy Body dementia –

According to most spouses and other family members the coach also motivated them to apply the intervention in daily life. Both spouses and other family members expressed they felt better equipped as caregiver and more prepared for the future. They felt the Partner in Balance intervention helped them to prioritize and adjust their expectations regarding their relative with YOD. In turn, some felt more able to cope with behavioral symptoms in their relative with YOD.

“I am especially more self-aware and Partner in Balance gave me the tools to tackle unwanted situations.”

– 58 year old spouse of a person with frontotemporal dementia –

Some participants expressed that the use of the chat function felt unnecessary, explaining why spouses provided on average a lower grade (Mean = 4.7, SD = 1.8) for the chat in the PPQ. An explanation stems from the fact that six of the 11 (54.5%) spouses received coaching from their own dementia casemanager or psychologist. In these cases, care as usual continued and most spouses also had in-person meetings with their coach.

### 3.2 Preliminary effects on role adaptation

**Fig. 4:**
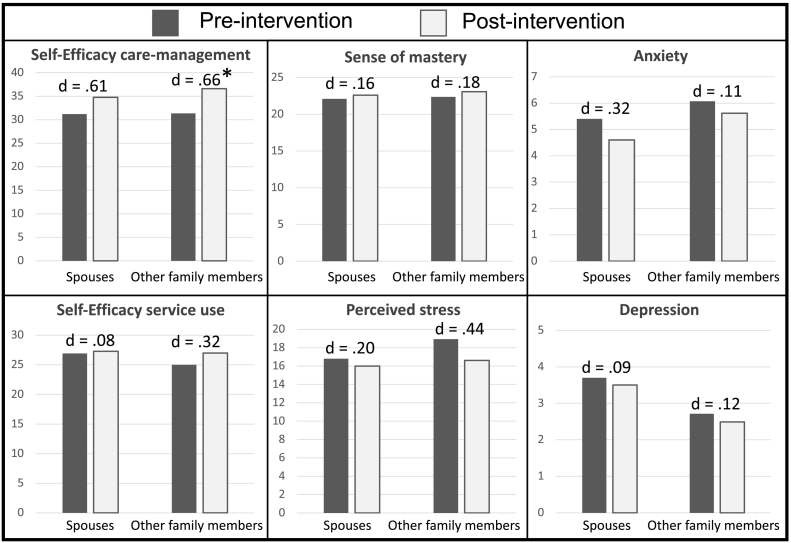
Scores on the pre- and post-questionnaire d = Cohen's d, * = statistically significant (*p* ≤ .05).

Eleven spouses and 14 other family members completed the questionnaire before and after participating in the intervention. Although all average scores increased or decreased in line with our hypothesis (Fig. 4), only the effect on self-efficacy regarding care-management was statistically significant in other family members, *t*(12) = 3.37, *p* = .006.

## 4 Discussion

The findings reveal that the tailored content for spouses and other family members of people with YOD was positively received. The participants valued the quality of the content provided and felt the intervention was easy to integrate in daily life. Similar to previous findings on generic modules included in Partner in Balance (Boots et al., 2018; Boots et al., 2016), the tailored content facilitated role adaption as participants felt better equipped for the caregiving role after the intervention. In both groups, caregivers expressed to feel more confident to cope with future challenges after participating in the intervention. In the end-user test with other family members, the effect on self-efficacy regarding care-management also increased statistically significant. This is in line with previously demonstrated effects of Partner in Balance in a randomized controlled trial (Boots et al., 2018). It is suggested that helping caregivers to become more resilient by increasing self-efficacy may also prevent long-term psychological distress and increase well-being (Bandura, 1997; Lazarus and Folkman, 1984).

Similar to previous studies, participants in our study felt the Partner in Balance intervention offered them a different perspective on their role as caregiver (Boots et al., 2018; Boots et al., 2016). Dementia is often defined in terms of loss and disability. As a result caregivers may perceive a lack of future perspective and experience feelings of hopelessness (Chapman et al., 2019; de Vugt and Droes, 2017). Providing caregivers with a perspective that acknowledges their loss but also encourages them to think in terms of possibilities may empower them and may help caregivers regain a sense of balance in combining their caregiving role with other roles and responsibilities (de Vugt and Verhey, 2013). This may particularly apply to YOD caregivers given challenges they experience to balance caregiving with family life, employment or studying (Cabote et al., 2015; van Vliet et al., 2010b). Therefore, it is important that interventions to support YOD caregivers can be easily integrated in daily life. Our findings reveal that Partner in Balance offered a flexible support tool that was easily integrated in daily life and allowed participants to follow the intervention at a convenient place and time. It is known that children of persons with YOD have specific support needs as they seem more reluctant to seek help from healthcare professionals (Millenaar et al., 2014). Additionally, children often feel that there is hardly any appropriate support available for them (Barca et al., 2014). Our findings indicate a web-based design is a particular good fit for children as some felt it was easier to share emotions online, compared to traditional face-to-face support. To further tailor Partner in Balance to the specific needs of children they suggested to improve the compatibility of the website with smartphone use. For example, to facilitate use in public transport.

Caregivers of persons with YOD often perceive main stream dementia services and support as age-inappropriate because such they primarily focused on elderly with dementia. In turn, YOD caregivers are known to delay the initiation of care and support services (Rabanal et al., 2018; Sikes and Hall, 2018). This is problematic because YOD can cause high levels of burden and distress in caregivers (Cabote et al., 2015; Millenaar et al., 2016b). Previously studies on Partner in Balance confirmed that YOD caregivers experienced difficulty recognizing their situation in the Partner in Balance intervention because most content focused on caregivers of elderly with dementia (Boots et al., 2017). Our findings show the newly incorporated content on YOD was positively received by YOD caregivers as they valued the high level of recognizable content provided. For example, in the video materials and narrative stories. However, our findings revealed that some caregivers of persons with FTD felt it was difficult to relate to the stories and content that mainly focused on YOD caused by AD. Caregivers of persons with FTD are known to experience a lack of understanding in caring for their relative, also from the low availability of recognizable support services (Bruinsma et al., 2020). Addressing their specific needs is important because FTD caregivers experience specific challenges that complicate adapting to the caregiving role. In particular, coping with profound changes in social behavior can impose high levels of burden and distress in FTD caregivers (Nunnemann et al., 2012).

### 4.1 Strengths and limitations

Our study aimed to evaluate the feasibility and usability of the proven effective Partner in Balance intervention in a new target group, and to identify directions for further improvement. Preliminary effects were also examined to evaluate if the intervention facilitated role adaptation, similar to previous findings (Boots et al., 2018; Boots et al., 2016). This end-user test with limited statistical power, shows a promising trend as absolute levels of self-efficacy and mastery increased, and levels of stress, anxiety and depression decreased but not statistically significant. Despite the small sample, self-efficacy in other family members did improve statistically significant. Increased self-efficacy may have a long-term beneficial effect on experienced stress, anxiety and depression (Bandura, 1997; Lazarus and Folkman, 1984). An explanation for not finding significant improvement on stress, anxiety and depression may stem in the fact that our study used a short follow-up with a small sample. Therefore, studying long-term effects using a larger sample remains a direction for future research.

A strength of our study is that qualitative and quantitative data was collected to evaluate how caregivers perceived the newly incorporated content. Additionally, a diverse sample was included that varied in age, gender, relationship to the person with YOD, and dementia subtype in their relative with YOD. A limitation is that experience with using web-based technology was not inventoried because this may influence how participants use Partner in Balance in daily life. In the interviews were no indicators found that participants perceived technological barriers. Given the young age of our sample (mean = 50.1, ranging from 17 to 74) most participants probably had experience with using web-based technologies in daily life, such as smartphones and computers.

During our study, usual care and support provided to informal caregivers by their casemanager or other healthcare professionals continued. To illustrate, some caregivers received coaching by their own healthcare professional. Previously, this showed to strengthen the bond between healthcare professionals and caregivers (Boots et al., 2017). Additionally, caregivers were allowed to receive other support such as peer-support. This may have biased our results, but it also resembles how Partner in Balance is used in the reality of daily practice.

### 4.2 Future directions and conclusion

In line with the results of the evaluation study of Partner in Balance for caregivers of people with late-onset dementia (Boots et al., 2018; Boots et al., 2016), the tailored intervention for YOD caregivers showed to be a good fit. Similar to earlier studies, the qualitative and quantitative measures indicate Partner in Balance has the potential to facilitate adaptation to the caregiving role. Our findings confirm that goal-setting is an important intervention element because it helped caregivers to translate the intervention to daily life. However, some caregivers experienced difficulty formulating specific, measurable and attainable goals during our study. Therefore, incorporating strategies in the intervention that further facilitate goal-setting may support caregivers with applying the intervention in daily life. As the coach proved an important source of inspiration, it may be helpful to embed an introduction on goal-setting at the start of the intervention to help caregivers with making goals more specific. Additionally, goal-setting may be facilitated by asking caregivers when and where they want to attain certain goal-directed behavior in the step-by-step change plan (Vohs and Baumeister, 2016). Incorporating specific content for caregivers of FTD patients would improve the fit with this specific subgroup of YOD, and fulfil their need for tailored interventions (Bruinsma et al., 2020).

Furthermore, an implementation strategy is required to sustain the Partner in Balance intervention because less than 3% of the dementia care and support interventions are implemented in daily practice (Gitlin et al., 2015). Therefore, the development of a business-model including a license agreement is underway to ensure that healthcare professionals can structurally work with the intervention (Christie et al., 2020). To facilitate the development of the business-model, a cost-effectiveness study seems a direction for the future to obtain insight in long-term benefits of the Partner in Balance intervention such as prevention of psychological problems in caregivers or delayed institutionalization of persons with dementia. It would be interesting to evaluate effects on long-term stress, anxiety, and depression. Additionally, it would be interesting to investigate how the effects of Partner in Balance could be enhanced on the long-term, and how the effects relate to other psycho-educational interventions or psychosocial support, such as peer-support or case management.

To persuade healthcare organizations to implement the intervention, we aimed to use end-user tests as a steppingstone to facilitate implementation from the start (Christie et al., 2019). In a subsequent phase, healthcare professionals are asked to see if they are willing to sustain working with the Partner in Balance intervention. As organizational sponsorship seems to be a facilitator of implementation (Christie et al., 2018), we aim to involve healthcare organizations affiliated with the Dutch Young-onset Dementia Knowledge Centre in the future implementation process.

Our findings demonstrate that tailoring support can help to make support services more appealing to caregivers of persons with YOD. Throughout the caregiving trajectory, YOD caregivers encounter a wide variety of supportive services, that often do not match their specific situation (Rabanal et al., 2018; Sikes and Hall, 2018). Partner in Balance is an addition to existing support services, by combining personal coaching and a web-based approach. However, this may not appeal to all caregivers of persons with YOD. Therefore, the development of other tailored supportive services remains an important direction for the future.

## Funding

This work was supported by an undisclosed funder of Alzheimer Research Fund Limburg; and partly performed during the UNited for Implementation of a healthCare standard In The Young (UNICITY)-project funded by 10.13039/501100001826ZonMw, [grant number: 60-63900-98-001].

## Declaration of competing interest

None.
